# Early-onset neonatal sepsis in a Chinese maternal and child healthcare centre, 2017–2023

**DOI:** 10.3389/fped.2025.1521908

**Published:** 2025-04-15

**Authors:** Weilin Wang, Zhenhui Wang, Linghua Wang, Suping Li, Jiangling Liu, Yaqin Huang, Jun Liu, Chun Zhu, Juan Zhang, Cairong Li, Chunjia Yang, Qiong Chen, Wenqin Wang, Shulin Deng, Yiming Du

**Affiliations:** ^1^ Department of Neonatology, Hunan Provincial Maternal and Child Health Care Hospital, University of South China, Changsha, Hunan, China; ^2^ Department of Obstetrics, Hunan Provincial Maternal and Child Health Care Hospital, University of South China, Changsha, Hunan, China; ^3^ Department of Clinical Laboratory, Hunan Provincial Maternal and Child Health Care Hospital, University of South China, Changsha, Hunan, China

**Keywords:** early-onset sepsis, incidence, antimicrobial resistance, surveillance, pathogens

## Abstract

**Objective:**

Early-onset sepsis (EOS) remains an important issue in neonatal units. Characteristics of EOS from China have not been fully revealed yet. Our aim is to investigate epidemiology, microbiology and clinical feature of EOS in a Chinese maternal and child healthcare centre.

**Methods:**

This is a retrospective observational study of EOS infants born in or admitted to Hunan Provincial Maternal and Child Health Care Hospital from January 1, 2017 to December 31, 2023.

**Results:**

During the study period, there were 131 neonatal infections. The incidence of EOS was 1.12 (95% CI 0.94–1.33) per 1,000 live births or 3.85 (95% CI 3.22–4.56) per 1,000 admissions. Coagulase-negative staphylococci (CoNS) (*n* = 43, 32.3%), group B *Streptococcus* (GBS) (*n* = 24, 18.0%) and *Escherichia coli* (*n* = 18, 13.5%) were the predominant pathogens. GBS screening test was performed before delivery in 77.7% mothers of all infants with EOS, and 12.9% of screening results were positive. Among the main pathogens causing EOS, 86% of CoNS strains were resistant to penicillin, while all GBS strains were susceptible to penicillin.

**Conclusions:**

We report a high burden of EOS among infants in the maternal and child healthcare centre from China. CoNS was the most frequent pathogen causing EOS. Longitudinal epidemiologic surveillance is required to improve empiric antibiotic treatment of EOS.

## Introduction

At present, developed countries such as the United States and the United Kingdom have established population-based surveillance network for bacterial infections among infants (1, 2). Monitoring the epidemiological characteristics of early-onset sepsis (EOS), such as incidence rate and pathogen distribution patterns, is essential to guide empirical antibiotic treatment in infants with suspected sepsis. With the implementation of management strategies for perinatal infections such as intrapartum antibiotic prophylaxis (IAP), the incidence of early-onset group B *Streptococcus* (GBS) disease has significantly decreased (3). In addition, the antimicrobial resistance (AMR) rate in neonatal units remains high. Recent application of antimicrobial stewardship programs in neonatal units showed the association with reduction in the initiation and duration of antimicrobial use (4).

Previous studies reported distinct patterns of pathogen distribution and clinical features in infection surveillance in different medical setting models (5, 6). Maternal and child healthcare centres are women and children specialized health facilities charged with basic labor services in China. These centres are first choice of pregnant women to labor, and provides basic medical services and physical examinations for women and children. They are classified as provincial, municipal and county-level. Our study gave a brief insight to epidemiology, microbiology and clinical feature of EOS in a provincial maternal and child healthcare centre from China.

## Methods

### Study design and population

This study retrospectively collected data on neonatal EOS from January 1, 2017 to December 31, 2023 in Hunan Provincial Maternal and Child Health Care Hospital. The microbiological and clinical data of neonates with EOS admitted to the neonatal unit, whether inborn (born in the hospital) or outborn (outside the hospital or in other hospitals), were involved in the study. Infants were transferred from the maternity unit to the neonatal unit when suspected sepsis. Our hospital is the largest maternal and child healthcare centre in Hunan province, China, and has 150 beds for neonatal hospitalization. The study was approved by the Ethics Committee of Hunan Provincial Maternal and Child Health Care Hospital (Fast no.2024037).

### Data collection and definitions

EOS was defined as a positive blood culture or cerebrospinal fluid (CSF) culture to a pathogen within 72 h after birth with clinical and laboratory ﬁndings consistent with infection. Cultures of Coagulase-negative staphylococci (CoNS) considered possible contaminants were excluded by clinical judgment (7). Antimicrobial susceptibility testing was followed by Clinical Laboratory Standards Institute (CLSI) guidelines (8). Data of demographics of infants and mothers, maternal risk factors, comorbidity and resource use in the neonatal unit of newborns and antibiotic resistance of main strains were recorded. Premature rupture of membranes was deﬁned as rupture of membranes prior to delivery. Clinical chorioamnionitis was defined by the presence of maternal fever >38.0°C during labor with at least two of the following criteria: uterine tenderness (without another cause), fetal tachycardia, maternal leukocytosis, maternal heart rate >100 bpm, foul-smelling amniotic ﬂuid (9). Bacterial meningitis was deﬁned as positive CSF culture, Gram staining, or neutrophilic leukocytosis, with or without low sugar (less than 50% of plasma glucose level) and high protein content in CSF samples. Septic shock was deﬁned as hypotension requiring catecholamine treatment (9).

### Statistical analysis

Data were analysed using Stata V.16 (Stata Corp., College Station, Texas, USA). The incidence rates were calculated as cases per 1,000 live births (by dividing the number of inborn infants with sepsis by the number of live births in the hospital) or cases per 1,000 admissions (by dividing the number of inborn and outborn infants with sepsis by the number of infants admitted to the neonatal unit), and the 95% conﬁdence intervals (CIs) for the incidence rates were calculated using the Poisson distribution. The incidence per 1,000 live births of a certain pathogen was calculated by dividing the number of inborn infants with the pathogen-caused sepsis by the number of live births in the hospital. The incidence of EOS by 1,000 live births was only calculated in inborn infants. The incidence of EOS by 1,000 admissions was calculated in infants admitted to the neonatal unit, inborn or outborn. The χ^2^ test was used to evaluate the differences in proportion, and χ^2^ for linear trend was used to test for trends in the incidence of EOS over the study period. *P* < 0.05 was considered signiﬁcant.

## Results

### Incidence of neonatal EOS

During the seven-year study period, a total of 116,044 live births were recorded in Hunan Provincial Maternal and Child Health Care Hospital. There were 130 cases of neonatal EOS occurred among inborn neonates, therefore the incidence was 1.12 (95% CI 0.94–1.33) per 1,000 live births. The incidence of EOS did not change over the study period (*p* = 0.085). After stratiﬁcation by gestational age and birth weight, the incidence of EOS was signiﬁcantly higher in neonates <32 weeks of gestation (*p* < 0.001) and <1,500 g (*p* < 0.001) (Table 1).

**Table 1:** Incidence of EOS among inborn neonates out of 1,000 live births, 2017–2023.

Neonates (LB = 116,044)	Years							
	2017	2018	2019	2020	2021	2022	2023	Total (95% CI)
Gestational age								
<32 weeks	6.29	0.0	0.0	7.19	10.64	21.13	14.60	8.31 (4.84–13.31)
32–36 weeks	1.75	1.69	2.27	0.72	0.65	4.02	1.32	1.79 (1.09–2.76)
≥37 weeks	0.58	1.16	0.80	1.05	0.66	1.18	0.91	0.90 (0.73–1.11)
Birth weight								
<1,500 g	15.54	0.0	0.0	11.70	8.73	16.06	8.66	8.29 (4.41–14.18)
1,500–2,499 g	1.57	2.87	2.10	0.0	2.42	5.82	3.47	2.62 (1.66–3.94)
≥2,500 g	0.56	1.06	0.84	1.09	0.57	1.22	0.88	0.89 (0.72–1.09)
Total	0.80	1.19	0.93	1.13	0.84	1.79	1.20	1.12 (0.94–1.33)

Results are given as n, cases per 1,000 live births. LB, Live Births.

The yearly incidence of EOS among inborn and outborn infants out of admissions is shown in Table 2. There were 131 neonatal infections, of which 130 (99.2%) occurred among inborn and one (0.8%) among outborn infants. The incidence of EOS for admissions was 3.85 (95% CI 3.22–4.56) per 1,000 admissions. The incidence of EOS was signiﬁcantly higher in very preterm infants (<32 weeks) (*p* < 0.001) and very low birth weight infants (<1,500 g) (*p* = 0.003). The incidence of EOS for admissions did not change over the study period (*p* = 0.329).

**Table 2:** Incidence of EOS among inborn and outborn neonates out of 1,000 admissions, 2017–2023.

Neonates (*N* = 34,055)	Years							
	2017	2018	2019	2020	2021	2022	2023	Total (95% CI)
Gestational age^a^								
<32 weeks	9.22	0.0	0.0	9.05	11.63	21.66	16.81	10.13 (5.90–16.22)
32–36 weeks	2.31	2.16	2.90	0.91	0.78	4.94	1.56	2.24 (1.37–3.46)
≥37 weeks	3.22	5.26	3.49	4.52	2.79	4.86	3.77	4.01 (3.24–4.91)
Birth weight^b^								
<1,500 g	17.54	0.0	0.0	10.75	15.04	15.56	9.01	8.74 (4.66–14.95)
1,500–2,499 g	1.64	3.09	2.35	0.0	2.67	6.52	3.63	2.83 (1.80–4.25)
≥2,500 g	3.09	4.79	3.65	4.67	2.35	4.91	3.57	3.89 (3.14–4.75)
Total	3.25	4.15	3.19	3.88	2.73	5.81	3.83	3.85 (3.22–4.56)

Results are given as *n*, cases per 1,000 admissions.

^a^ Data of gestational age was not available in 1 patient.

^b^ Data of birth weight was not available in 5 patients.

### Pathogen distribution

A total of 133 isolates were identiﬁed in 131 infants admitted to the neonatal unit (Table 3). Gram-positive bacteria accounted for 93 (69.9%) of 133 isolates. The most frequent pathogens were CoNS (*n* = 43, 32.3%), GBS (*n* = 24, 18.0%) and *Escherichia coli* (*n* = 18, 13.5%). Among these, 132 pathogens were isolated from inborn neonates. The incidence of CoNS EOS was 0.37 per 1,000 live births, while the incidence of GBS and *E. coli* EOS was 0.21 and 0.16 per 1,000 live births.

**Table 3:** Pathogen distribution of EOS.

Pathogens	Proportion, *n* (%) (*N* = 133)	Incidence, cases per 1,000 births (95% CI) (LB = 116,044)
Gram-positive bacteria	93 (69.9)	0.80 (0.65–0.98)
Coagulase-negative staphylococci	43 (32.3)	0.37 (0.27–0.50)
Group B *Streptococcus*	24 (18.0)	0.21 (0.13–0.31)
*Staphylococcus aureus*	8 (6.0)	0.07 (0.03–0.14)
*Streptococcus* spp.	8 (6.0)	0.07 (0.03–0.14)
*Enterococcus* spp.	7 (5.3)	0.06 (0.02–0.12)
Other gram-positive bacteria^a^	3 (2.3)	0.03 (0.005–0.08)
Gram-negative bacteria	39 (29.3)	0.34 (0.24–0.46)
*Escherichia coli*	18 (13.5)	0.16 (0.09–0.25)
*Acinetobacter* spp.	5 (3.8)	0.04 (0.01–0.10)
*Stenotrophomonas maltophilia* ^c^	5 (3.8)	0.03 (0.01–0.09)
Other gram-negative bacteria^b^	11 (8.3)	0.09 (0.05–0.17)
Fungi	1 (0.8)	0.01 (0.0002–0.05)
*Candida* spp.	1 (0.8)	0.01 (0.0002–0.05)

LB, Live Births.

^a^ Including two cases of *Listeria monocytogenes* and one case of *Aerococcus viridans*.

^b^ Including two cases of *Moraxella* spp., two cases of *Burkholderia cepacia*, two cases of *Serratia* spp., one case of *Ochrobactrum anthropi*, one case of *Alcaligenes* spp., one case of *Citrobacter* spp., one case of *Klebsiella* spp. and one case of *Pseudomonas* spp.

^c^ One case of *Stenotrophomonas maltophilia* was outborn and not included in incidence calculation.

The online Supplementary Figure S1 shows the spectrum of pathogens causing EOS according to gestational age. CoNS was the predominant pathogen in infants <32 weeks, 32–36 weeks and ≥37 weeks, accounting for 4 (23.5%), 7 (35%), 32 (33.3%) infections separately. The online Supplementary Table S1 describes the clinical characteristics of infants developing CoNS-induced sepsis. CoNS has been classified as *Staphylococcus epidermidis, Staphylococcus hominis, Staphylococcus capitis* and other CoNS. *E. coli* accounted for 4 (23.5%) infections in very preterm infants. GBS accounted for 24 (25%) infections in term infants. One case of *Candida glabrata* was reported in infants <32 weeks. Three (15%) cases of *Acinetobacter baumannii* were reported in infants born at 32–36 weeks. *Staphylococcus aureus* accounted for two infections in neonates at 32–36 weeks and six in neonates ≥37 weeks. *Stenotrophomonas maltophilia* accounted for one infection in neonates <32 weeks, one in neonates at 32–36 weeks and three in neonates ≥37 weeks.

### Characteristics of mothers and neonates

The characteristics of mothers and infants are described according to gestational age in Table 4. 80% mothers of term infants gave birth through vaginal delivery. GBS screening test was performed in 77.7% mothers of all infants and 77.7% mothers of term infants, in which 12.9% and 12.3% were positive results, respectively. Ten (58.8%) mothers of neonates <32 weeks developed clinical chorioamnionitis, with four of them leading to neonatal *E. coli* infection. A total of 59 (45.4%) women received intrapartum antibiotic use, mostly piperacillin (14/59, 23.7%) or second-generation cephalosporins (29/59, 49.2%).

**Table 4:** Maternal and neonatal characteristics of infants with EOS according to gestational age.

Characteristics	<32 weeks (*n* = 17)	32–36 weeks (*n* = 20)	≥37 weeks (*n* = 94)	Total (*N* = 131)
Birth weight, g, mean (SD)	1,333 (302)	2,168 (590)	3,267 (470)	2,857 (845)
Female, *n* (%)	6 (35.3)	9 (47.4)	43 (45.3)	58 (44.3)
Apgar ≥7 at 1 min, *n* (%)	14 (82.4)	19 (100.0)	91 (96.8)	124 (95.4)
Mother's age, years, mean (SD)	32.4 (5.0)	30.6 (4.8)	29.9 (3.7)	30.3 (4.1)
Method of delivery, *n* (%)				
Vaginal	8 (47.1)	5 (26.3)	76 (80.0)	89 (67.9)
Caesarean section	9 (52.9)	14 (73.7)	19 (20.0)	42 (32.1)
PROM, *n* (%)	10 (58.8)	9 (47.4)	32 (33.7)	51 (38.9)
Clinical chorioamnionitis, *n* (%)	10 (58.8)	2 (10.5)	9 (9.5)	21 (16.0)
Antenatal GBS screening, *n* (%)	15 (88.2)	13 (68.4)	73 (77.7)	101 (77.7)
Positive	1 (6.7)	3 (23.1)	9 (12.3)	13 (12.9)
Negative	14 (93.3)	10 (76.9)	64 (87.7)	88 (87.1)
Intrapartum antibiotic use, *n* (%)	13 (76.5)	15 (78.9)	31 (33.0)	59 (45.4)
Comorbidity, *n* (%)				
Bacterial meningitis	0 (0.0)	3 (15.8)	3 (3.2)	6 (4.6)
Intracranial hemorrhage	10 (58.8)	3 (15.8)	5 (5.3)	18 (13.7)
Septic shock	4 (23.5)	2 (10.5)	9 (9.5)	15 (11.5)
Invasive mechanical ventilation, *n* (%)	12 (70.6)	3 (15.8)	12 (12.6)	27 (20.6)
PN use, *n* (%)	17 (100.0)	17 (89.5)	41 (43.2)	75 (57.3)
Length of stay, days, median (IQR)	42 (22.5–69.5)	16 (14–21)	12 (10–14)	12 (11–17)
Transfer to another hospital, *n* (%)	1 (5.9)	1 (5.3)	4 (4.2)	6 (4.6)
Death, *n* (%)	4 (23.5)	1 (5.3)	0 (0.0)	5 (3.8)

PROM, premature rupture of membranes; GBS, group B *Streptococcus*; PN, parenteral nutrition.

Data was not available in 1 infant (Apgar); 1 infant (GBS screening); 1 infant (Intrapartum antibiotic use).

The most frequent antibiotic use of initial treatment of infants were piperacillin (54/131, 41.2%) or third-generation cephalosporins (21/131, 16.0%). The median length of stay in neonatal unit was 12 (11–17) days. Six infants (4.6%) were transferred to another hospital for further treatment. Of the 131 infants with EOS, five (3.8%) died during hospitalization, including four born <32 weeks and one born at 32–36 weeks.

### Antimicrobial resistance

Table 5 shows the AMR pattern of the main pathogens causing EOS, including CoNS, GBS and *E. coli*. Resistance to penicillin and ampicillin were found in 86% and 100% CoNS strains, respectively. All GBS strains were susceptible to penicillin. Among the eight *E. coli* isolates tested, three (37.5%) were resistant to ceftriaxone. No *E. coli* strain was found resistant to meropenem. Two of eight (25%) *S aureus* strains were identified as methicillin-resistant *Staphylococcus aureus* (MRSA).

**Table 5:** AMR pattern of the main organisms causing EOS.

Antibiotics	CoNS, *n*/*N* (%)	GBS, *n*/*N* (%)	*E.coli*, *n*/*N* (%)
Penicillin	37/43 (86.0)	0/24 (0)	0/0 (−)
Ampicillin	11/11 (100.0)	0/23 (0)	16/18 (88.9)
Amoxicillin/clavulanic acid	18/34 (52.9)	0/8 (0)	0/10 (0)
Piperacillin/tazobactam	10/23 (43.5)	0/0 (0)	2/18 (11.1)
Cefazolin	10/23 (43.5)	0/8 (0)	6/18 (33.3)
Cefuroxime	10/23 (43.5)	0/0 (0)	2/8 (25.0)
Ceftriaxone	10/23 (43.5)	0/24 (0)	3/8 (37.5)
Cefepime	0/0 (−)	0/6 (0)	2/18 (11.1)
Erythromycin	23/43 (53.5)	20/24 (83.3)	0/0 (−)
Clindamycin	10/42 (23.8)	18/24 (75.0)	0/0 (−)
Trimethoprim/sulfamethoxazole	5/43 (11.6)	0/0 (−)	7/18 (38.9)
Levofloxacin	6/31 (19.4)	7/24 (29.2)	4/18 (22.2)
Gentamicin	11/43 (25.6)	0/0 (−)	3/18 (16.7)
Amikacin	0/11 (0)	0/0 (−)	0/16 (0)
Meropenem	10/23 (43.5)	0/24 (0)	0/18 (0)
Tetracycline	3/43 (7.0)	19/21 (90.5)	7/10 (70.0)
Linezolid	0/43 (0)	0/24 (0)	0/0 (−)
Vancomycin	0/43 (0)	0/24 (0)	0/0 (−)

*n*, number of resistant isolates; *N*, number of tested.

## Discussion

We firstly provide the incidence of neonatal EOS in China by cases per 1,000 live births or admissions in detail, due to the convenience to reach the data on live births as a maternal and child healthcare centre. Furthermore, we also firstly show the epidemiology, microbiology and clinical feature of EOS in a model of maternal and child healthcare centre, which provides basic medical services and physical examinations for women and children. The pattern of pathogen distribution and AMR rate may be a guidance for clinical management of newborns at high risk of early-onset neonatal infection in the rooming-in ward of the maternity unit or neonatal unit in similar medical model in developing countries with high birth rates.

Al-Taiar et al. (10) reported an incidence of EOS of 0.62 (95% CI 0.45–0.82) per 1,000 live births or 4.91 (95% CI 4.22–5.68) per 1,000 admissions in a prospective cohort study between 2006 and 2009 in neonatal care units in China, Malaysia, Hong Kong and Thailand. Comparatively, our results showed a higher incidence of 1.12 (95% CI 0.94–1.33) per 1,000 live births but a lower incidence of 3.85 (95% CI 3.22–4.56) per 1,000 admissions. The incidence of EOS was also reported 0.78 per 1,000 live births (95% CI 0.61–0.97) in a retrospective study from South China (11). Therefore, we showed a high burden of EOS among infants in the maternal and child healthcare centre in China. Consistent with our results, several studies reported the difference in incidence of EOS according to gestational age or birth weight (9, 12, 13).

In a prospective surveillance study in the Paris area, France from 2019 to 2021, Sikias et al. (13) revealed the incidence of EOS of different pathogen infection in neonates born at ≥34 weeks of gestation. In our study, GBS infections were all detected from term infants. The incidence of GBS EOS was 0.21 (95% CI 0.13–0.31) per 1,000 live births. We also showed that CoNS was the predominant pathogen in neonatal EOS. CoNS was reported the main pathogen responsible for neonatal sepsis in neonatal units from China or other countries, especially in regions with a low prevalence of MRSA using an aminoglycoside-beta-lactam antibiotic combination as the ﬁrst-line empirical antimicrobial regimen to cover Gram-negative bacteria and methicillin-sensitive *S. aureus* (MSSA) (14). In our study, the clinical outcomes of neonates with CoNS infection were mostly good. CONS are not so virulent as Gram-negative bacteria and fungi, and CoNS infection was associated with the lower rate of short-term infectious complications as well as mortality. However, CONS are capable to exert a long-term detrimental effect on the host, particularly on infants <1,000 g (15). In our report, we also raise the awareness of CoNS infection in neonatal EOS. Further study should be considered in investigating the role of CONS in EOS.

The Chinese expert consensus for prevention of perinatal GBS disease was first issued in 2021 (16). The consensus standardized the timing and detection methods of GBS screening, and intrapartum antibiotic prophylaxis. The Neonatal Health Care Committee of Chinese Maternal and Child Health Association developed another expert consensus on the clinical management of newborns at high risk of early-onset neonatal infection in the rooming-in ward of the maternity unit (17). Our study described the antenatal risk factors of infants with EOS at <32 weeks, 32–36 weeks and ≥37 weeks. GBS screening test was performed in 77.7% mothers of infants with EOS, and 45.4% of the mothers received intrapartum antibiotic use. The practice of generalization of GBS screening and peripartum antibiotic prophylaxis is being performed in our centre in recent years. We would monitor and assess the effect of such practice in future studies.

Zhang et al. (18) reviewed the rates of AMR patterns for bloodstream isolates from Chinese neonates. We firstly provide the AMR pattern in the maternal and child healthcare centre in China. Recent Chinese consensus on management of neonatal sepsis recommends ampicillin combined with third-generation cephalosporin as first-line initial antibiotic use (19). However, our results showed high resistance rate to ampicillin and ceftriaxone in CoNS and *E. coli* strains. It might raise particular concern of considering novel combination of initial treatment for recommendation.

The strength of this study is the generalization of the EOS rate by cases per 1,000 live births or admissions. The incidence was less reported in previous studies of Chinese neonates, because most patients were admitted to specialized children's hospitals which lack of data on live births. We have provided data of seven years since the electronic medical recording system was introduced. The limitation of the study is mainly the retrospective design. In addition, multi-centre cooperation is needed for further regional or national surveillance in EOS infants.

In conclusion, the incidence of EOS was 1.12 per 1,000 live births or 3.85 per 1,000 admissions. CoNS, GBS and *E. coli* were the most common pathogens. The incidence of GBS EOS was 0.21 per 1,000 live births. Our data encourage the generalization of GBS screening, and raise the concern of CoNS infection in neonatal EOS. Longitudinal surveillance is required to monitor pathogen distribution and AMR pattern for adjusting EOS empiric treatment in initial management of infants with suspected infection.

## Data Availability

The raw data supporting the conclusions of this article will be made available by the authors, without undue reservation.
