# Supplementary material for: Early-onset neonatal sepsis in a Chinese maternal and child healthcare centre, 2017–2023
Source: Front Pediatr. 2025 Apr 15;13:1521908. doi: 10.3389/fped.2025.1521908 (PMC12037611; doi:10.3389/fped.2025.1521908)
Supplement: Supplementary file 1 [file Table1.docx]

Table S1. Clinical characteristics of infants developing CoNS-induced sepsis

| Characteristics | *Staphylococcus epidermidis* (n=25) | *Staphylococcus hominis* (n=5) | *Staphylococcus capitis* (n=4) | Other CoNS* (n=9) | Total (N=43) |
| --- | --- | --- | --- | --- | --- |
| Female | 15 (60.0) | 2 (40.0) | 2 (50.0) | 2 (22.2) | 21 (48.8) |
| Gestational age |  |  |  |  |  |
| <32 wk | 1 (4.0) | 0 (0.0) | 2 (50.0) | 1 (11.1) | 4 (9.3) |
| 32-36 wk | 5 (20.0) | 0 (0.0) | 1 (25.0) | 1 (11.1) | 7 (16.3) |
| ≥37 wk | 19 (76.0) | 5 (100.0) | 1 (25.0) | 7 (77.8) | 32 (74.4) |
| Birth weight |  |  |  |  |  |
| <1500 g | 1 (4.0) | 0 (0.0) | 2 (50.0) | 1 (11.1) | 4 (9.3) |
| 1500-2499 g | 6 (24.0) | 0 (0.0) | 0 (0.0) | 2 (22.2) | 8 (18.6) |
| ≥2500 g | 18 (72.0) | 5 (100.0) | 2 (50.0) | 6 (66.7) | 31 (72.1) |
| Method of delivery |  |  |  |  |  |
| Vaginal | 18 (72.0) | 3 (60.0) | 3 (75.0) | 4 (44.4) | 28 (65.1) |
| Caesarean section | 7 (28.0) | 2 (40.0) | 1 (25.0) | 5 (55.6) | 15 (34.9) |
| Apgar ≥7 at 1 min | 24 (96.0)** | 5 (100.0) | 4 (100.0) | 9 (100.0) | 42 (97.7) |
| Transfer to another hospital | 0 (0.0) | 0 (0.0) | 0 (0.0) | 0 (0.0) | 0 (0.0) |
| Death | 1 (4.0) | 0 (0.0) | 0 (0.0) | 0 (0.0) | 1 (2.3) |

Results are given as n (%).

*Including two cases of *Staphylococcus saprophyticus*, two cases of *Staphylococcus cohnii*, one case of *Staphylococcus schleiferi*, one case of *Staphylococcus simulans*, one case of *Staphylococcus haemolyticus*, one case of *Staphylococcus lentus* and one case of *Staphylococcus intermedius*.

**Data was not available in 1 infant (Apgar).
