# Supplementary figures and images for: Early-onset neonatal sepsis in a Chinese maternal and child healthcare centre, 2017–2023
Source: Front Pediatr. 2025 Apr 15;13:1521908. doi: 10.3389/fped.2025.1521908 (PMC12037611; doi:10.3389/fped.2025.1521908)

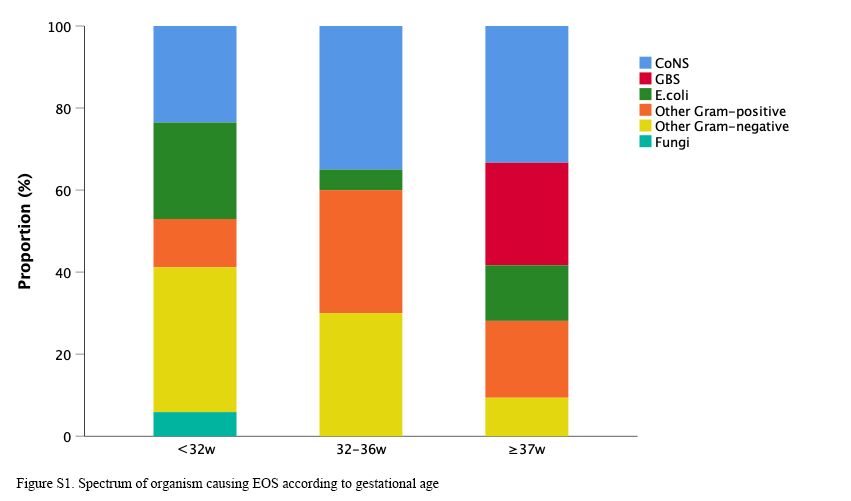

Supplement: Supplementary file 2 [file Image1.tif]
